# Its Attractions and Prospects

**Published:** 1920-09-04

**Authors:** 


### GENERAL PRACTICE.

### Its Attractions and Prospects.

The word "nationalisation" is one of the modern shibboleths. In the last twenty or thirty years the State has developed and extended a paternal interest in the welfare of its citizens, and the correct pronunciation of the word "nationalisation" has become a matter of great importance. The health of the community is regarded as the highest good; it is natural, therefore, that the influence of nationalisation should spread to medicine. The Labour party, notwithstanding its active interference in foreign politics, has found time to consider its future relationship with the medical profession, and has supported the nationalisation of the treatment of tuberculosis, of venereal disease, of the ailments of school children and of employed persons. The establishment of a Ministry of Health presages further developments in State organisation of health matters; and the reports of the Medical Consulative Councils of the Ministry are concerned with the elaboration of methods by which the State may introduce a systematised medical service for the community.

### The Medico-Political Ferment.

Many hundreds of young men and women will shortly be entering on their career as students of medicine; and in five years' time some of them will be receiving the State hall-mark of registration as persons competent to give medical advice and treatment to the people. The majority of those registered will become general practitioners; and it is interesting to speculate how far the present medico-political ferment is likely to alter the relationship of the general practitioner to his patients.

The pursuit of the ideal is a costly process; and the path of human progress is strewn with the wrecks of shattered hopes. As a rule, the catastrophe is due to the fact that little or no inquiry has been made into the nature and foundations of existing structures; a defect has been noticed; its relation to the rest of the edifice is not studied, a large hole is made in the building, and the excrescence put in its place fails to satisfy expectation. And in the construction of the excrescence little or no attention is paid to human nature.

It will be well, then, in the first place to consider the present position of the general practitioner; secondly, to indicate the defects from which general practice suffers, and, thirdly, to endeavour to forecast the conditions under which, five years hence, the general practitioner will work.

### The Cry for State Institutions.

Until recently the relation of general practitioner to patient was one of free contract between two individuals. The patient selected the doctor he wished, the doctor accepted the patient; and the fee for attendance was a matter of mutual arrangement. When the means of the patient did not permit of this arrangement the situation was met in various ways. The patient could obtain treatment for nothing in some charitable institution, such as a voluntary hospital, or he could become a Poor-law patient, or he could join a club or a friendly society and obtain treatment at what were called contract rates. The basis of these methods was a voluntary system, except in the case of the Poor-law, in which provision was made by the State. Objections, however, were raised to all these methods. The voluntary hospital system was a charity; and the leaders of the Labour party prefer that charity should be camouflaged by being paid for by the State. The friendly society was in theory an excellent institution, but, owing to the cheeseparing of contributions for medical attendance by the societies, and the slackness of doctors in failing to assert their right to a predominant position in the societies, the system became discredited. The Poor-law system involved what is now known as the stigma of pauperism; the public forgets that by whatever name it is called, pauperism is in the majority of cases the result of lack of thrift. For these reasons there arose a demand for the State provision of medical treatment, just as the State had already taken over the provision of education.

The Insurance Act was the method by which the demand was met. It was soon found, however, that the Insurance Act failed to satisfy the whole demand. National Insurance only applies to a minority of the population; it has not attracted all the best doctors, it only provides such treatment as is regarded as being within the competence of the general practitioner. The advances in medicine and surgery are considered to require greater facilities in equipment, greater knowledge and skill than are within the reach of the ordinary practitioner. Hence the development of the schemes outlined in the reports of the Consultative Councils of the Ministry of Health. Notwithstanding the comparatively high standard of health in this country, there is a good deal of truth in many of the alleged defects in health matters. Although many of these defects could be made good by voluntary effort, and although there are occasional signs of reaction against excessive State paternalism, it seems impossible to suppose that the State, having embarked on a career of interest in the health of the community, will now draw back and leave health matters to voluntary efforts.

### Nine-tenths Panel Patients.

What then will be the conditions of general practice a few years hence? Probably there will be a State service, based on the panel system, open to all who choose to avail themselves of it. Nominally a charge of some sort will at first be made to the patients—possibly on some graduated scale. But the difficulty of working such a system will lead in time to the abolition of fees, just as in the case of educa-

**General Practice**—(*continued*).

tion. Those who wish to do so will be able to consult their doctor as at present, paying his private fees, and obtaining in return a treatment which will be regarded as superior because it is paid for. The great difficulty which the State will have to face is the panel practitioner in a single-handed area. At present the man who accepts a patient on his panel is unable to treat that patient privately. But if anything like the panel system is extended to the dependents of panel patients nine-tenths of the population will come under the system. Moreover, the Dawson Report contemplates every person, irrespective of station, who is seriously ill, being entitled to hospital treatment at health centres. Therefore, nine-tenths of the population in a single-handed country district will be unable to see the doctor except as panel patients, and the other tenth may have the option of State treatment. It seems inconceivable that the public will surrender its freedom to this extent; it is more probable that, with a service open and free to all, the State will have to abandon its present attitude, and restore a measure of freedom to the public. The members of the community will themselves decide whether they shall avail themselves of what the State offers, or obtain what they require at their own cost.

A Reaction Against State Paternalism.

In any case it seems impossible to avoid a definite division into two kinds of practice, a division which is already seen as a result of the Insurance Act, and which is unavoidable when the State begins to interfere. In such a position there is nothing to prevent good work being done. The State teacher does good work as well as the teacher in a great public school. But as long as human nature remains what it is, as long as a self-respecting person likes to feel that he is self-supporting and wishes to feel dependent on other people to the smallest degree that he can manage, so long will a system of private practice remain. And a large part of the public will aim at being able to pay their own doctors, while the majority of doctors will aim at freeing themselves from State control. To the young man the State system will offer work at the very time when he most needs it. The older man well-established will leave this work to the younger. Whether this will hold when the younger man becomes the older may be a matter of doubt. But perhaps it is expected that by that time Socialism in its fullest sense will have become an established fact and that the State will allot so many patients to each doctor, and will compel so many patients to be treated by the doctor it selects for them. In the meantime the new medical student may be of good cheer, confident that paths for useful work will be open to him even if the day of reaction against State paternalism is not yet in sight. So long as money remains the medium of exchange, so long will private practice as at present understood remain an established system.

